# Locus Coeruleus to Paraventricular Thalamus Projections Facilitate Emergence From Isoflurane Anesthesia in Mice

**DOI:** 10.3389/fphar.2021.643172

**Published:** 2021-04-27

**Authors:** Yawen Ao, Bo Yang, Caiju Zhang, Bo Wu, Xuefen Zhang, Dong Xing, Haibo Xu

**Affiliations:** Department of Radiology, Zhongnan Hospital of Wuhan University, Wuhan University, Wuhan, China

**Keywords:** locus coeruleus, paraventricular thalamus, general anesthesia, emergence, optogenetics and DREADDs

## Abstract

Locus coeruleus (LC) sends widespread outputs to many brain regions to modulate diverse functions, including sleep/wake states, attention, and the general anesthetic state. The paraventricular thalamus (PVT) is a critical thalamic area for arousal and receives dense tyrosine-hydroxylase (TH) inputs from the LC. Although anesthesia and sleep may share a common pathway, it is important to understand the processes underlying emergence from anesthesia. In this study, we hypothesize that LC TH neurons and the TH:LC-PVT circuit may be involved in regulating emergence from anesthesia. Only male mice are used in this study. Here, using c-Fos as a marker of neural activity, we identify LC TH expressing neurons are active during anesthesia emergence. Remarkably, chemogenetic activation of LC TH neurons shortens emergence time from anesthesia and promotes cortical arousal. Moreover, enhanced c-Fos expression is observed in the PVT after LC TH neurons activation. Optogenetic activation of the TH:LC-PVT projections accelerates emergence from anesthesia, whereas, chemogenetic inhibition of the TH:LC-PVT circuit prolongs time to wakefulness. Furthermore, optogenetic activation of the TH:LC-PVT projections produces electrophysiological evidence of arousal. Together, these results demonstrate that activation of the TH:LC-PVT projections is helpful in facilitating the transition from isoflurane anesthesia to an arousal state, which may provide a new strategy in shortening the emergence time after general anesthesia.

## Introduction

General anesthesia (GA) is a reversible state of unconsciousness induced by various kinds of general anesthetics (Brown et al., 2010; Brown et al., 2011). Achieving a controllable and smooth emergence from general anesthesia is of particular importance for surgical patients. Traditionally, emergence from general anesthesia has been treated as a purely passive process. However, accumulating evidence suggest that emergence from anesthesia is also an active and controllable process (Kelz et al., 2019). Rencently, there has been more interests on actively and rapidly inducing emergence from GA (Tarnal et al., 2016; Kelz et al., 2019).

The locus coeruleus (LC), a brainstem pontine nucleus of noradrenergic neurons, plays a critical role in core behavioural and physiological processes (Chandler et al., 2019; Poe et al., 2020). Recent studies have illustrated how the complex efferent system of LC neurons selectively mediates specific behaviours (Berridge and Waterhouse, 2003; Reyes et al., 2008; Li et al., 2018). It has been reported that LC TH population promotes immediate sleep-to-wake transitions (Carter et al., 2010). However, it remains to be defined whether LC TH neurons participate in anesthesia arousal.

The paraventricular thalamus (PVT), the main component of the dorsal thalamic midline, has been implicated in chronic stress, emotion and arousal (Colavito et al., 2015; Hua et al., 2018; Ren et al., 2018). PVT neurons are primarily excitatory neurons (Frassoni et al., 1997). A recent study has shown that lesion of the PVT causes fragmentation of wakefulness and that the optical stimulation of PVT glutamatergic neurons during sleep induces transitions to wakefulness (Ren et al., 2018). This study also shows that activation of PVT neurons induce an acceleration of emergence from general anesthesia. Considered together, both LC and PVT are involved in the anesthesia arousal. These data thus support that both LC and PVT might be involved in the anesthesia arousal, a circumstance that has not been experimentally demonstrated yet.

Given that retrograde neural tracing confirmed the anatomical TH:LC-PVT projection (Beas et al., 2018), we hypothesized that TH:LC-PVT projections played an important role in anesthesia arousal. In this study, we found that LC TH neurons were active during passive emergence from isoflurane anesthesia. Chemogenetic activation of LC TH neurons elicited behavioral and cortical arousal from isoflurane anesthesia. Furthermore, c-Fos expression increased in the PVT after LC TH neurons activation. Optogenetic excitation of the TH:LC-PVT projections promoted cortical arousal and emergence from anesthesia. In addition, chemogenetic inhibition of this circuit prolonged emergence process. Thus, these results reveal that the TH:LC-PVT projections play a critical role in accelerating anesthesia emergence in rodents.

## Materials and Methods

### Animals

The guidelines of the National Research Council Guide for the Care and Use of Laboratory Animals were strictly followed throughout the experiment. Wild type C57BL/6J male mice and tyrosine hydroxylase (TH)-internal ribosome entry site (IRES)-Cre in heterozygous male mice (8–12 w) weighing 25–30 g were used for all experiments and randomly assigned to groups. All mice were given food and water *ad libitum* with a controlled temperature (22 ± 2°C) under a 12 h/12 h light/dark cycle (lights on between 7:00 and 19:00). All efforts were made to minimize the number of mice used. All mice were acclimated to the animal facility at least 3 days before the initiation of the experiments. To minimize the influence of the diurnal rhythm of sleep-wakefulness on results, all experiments were performed between 10:00 and 19:00. We chose to perform the experiment between 10:00 and 19:00 because PVT neurons display state-dependent activity patterns with the highest activity shown during the hours of darkness (Ren et al., 2018). Thus, we conducted the experiments when the activity of PVT neurons is relatively low. All animal experiments were conducted according to protocols approved by the Institute of Animal Care Committee at Zhongnan Hospital of Wuhan University.

### Surgery

Mice were anesthetized with pentobarbital sodium and placed in a stereotaxic apparatus (Narishige, Japan). Adeno-associated viruses (AAVs, BrainVTA Technology Co., Ltd., China) were injected bilaterally into the LC (coordinates, bregma: AP = −5.26 mm; ML = ± 1.05 mm; DV = −3.75 mm, 200 nl for each site). After each injection, the glass micropipette was kept in place for 5 min to ensure sufficient virus diffusion. All viruses were sufficiently expressed for at least 3 w before the experiments. Optical fibers (diameter: 200 μm, Coocore Inc., China) were implanted above the PVT (coordinates, bregma: AP = −1.1 mm; ML = +0.55 mm; DV = −2.8 mm, with a 10° angle towards the midline). For chemogenetic inhibition experiments, a guide cannula (OD, 0.48 mm; ID, 0.34 mm; length, 3.5 mm; RWD) was implanted above the PVT (coordinates, bregma: AP = −1.1 mm; ML = +0.55 mm; DV = −2.75 mm, with a 10° angle towards the midline). Two stainless steel screw electrodes was implanted on the skull surface (coordinates, recording electrode, bregma: AP = +1.75 mm; ML = −0.4 mm; reference electrode: cerebellum). The coordinates were measured from bregma according to The Mouse Brain in Stereotaxic Coordinates (Paxinos and Franklin, 2004). The EEG electrodes were affixed to the skull with Super-Bond C&B and dental acrylic. After surgery, mice were allowed to recover for at least 1 w before the experiments.

### Experiment Protocol

#### c-Fos Expression in the LC After Exposure to Isoflurane With or Without Emergence

**FIGURE 1:**
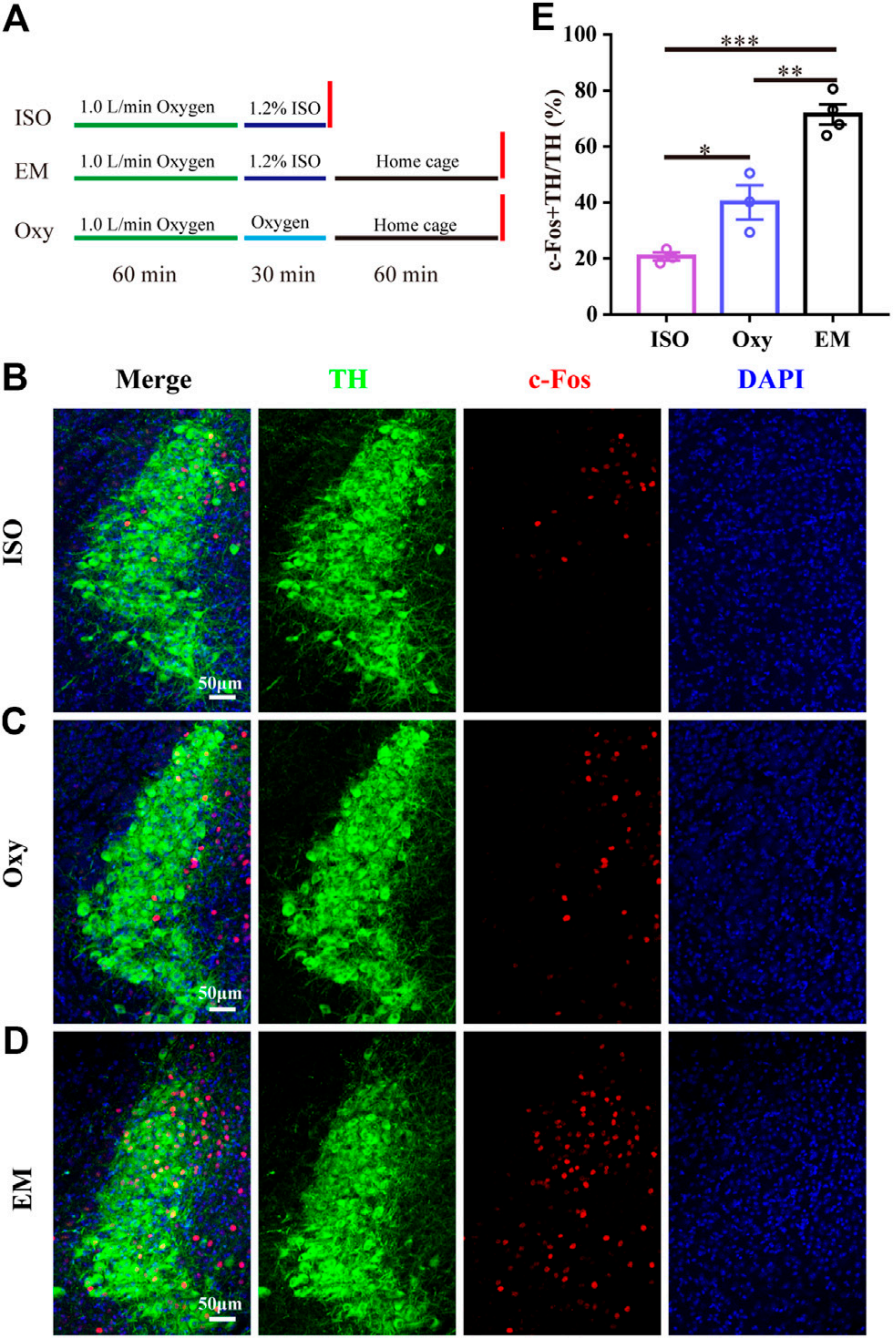
LC TH neurons are active during passive emergence from isoflurane anesthesia. **(A)** The experimental protocol for the c-Fos quantification experiments in three groups of mice. First group (ISO) received 1.2% isoflurane anesthesia and was euthanatized before awakening. The second group (EM) received the same isoflurane anesthesia treatment and underwent passive emergence from anesthesia. The third group (Oxy) did not underwent isoflurane anesthesia and received oxygen. **(B–D)** Representative photomicrographs showing the co-localization of c-Fos (red) and TH neurons (green) in LC for ISO group, Oxy group and EM group, respectively. **(E)** The percentage of c-Fos + TH/TH in ISO group (*n* = 3), Oxy group (*n* = 3) and EM group (*n* = 4). Data were analyzed by one-way ANOVA with post hoc Bonferroni multiple-comparison test, *p < 0.05, **p < 0.01, ***p < 0.001. Data shown as means ± SEM.

C-Fos immunohistochemistry was used to determine the activity of the LC TH neurons during emergence from isoflurane anesthesia. The time course of the c-Fos expression experiment is schematized in Figure 1A. Briefly, one group of mice was exposed to 30 min 1.2% isoflurane (ISO) anesthesia protocol and sacrificed immediately at the end of the anesthesia. Another group of mice was also exposed to the anesthesia protocol for 30 min and then allowed to emerge (EM) in their home cage, 60 min later mice were sacrificed. The last group of mice was exposed only to 100% oxygen (Oxy) for 30 min and then sacrificed 60 min later. The isoflurane concentration was monitored with Drager Vamos Plus Anesthesia Monitor (Dräger, Germany) and maintained constant through the experiment.

#### Chemogenetic Activation of LC TH Neurons Experiment

AAV-hSyn-DIO-hM3Dq-mCherry or AAV-EF1a-DIO-EYFP was injected bilaterally into the LC of TH-Cre mice. Clozapine N-oxide (CNO, dissolved in saline, Enzo Life Science Inc., United States) was administered intraperitoneally (i.p.) to activate the hM3Dq receptors 1 h before isoflurane infusion. Saline treatment was used as control. We employed the c-Fos, a neural activity marker (Carter et al., 2017), to verify that the LC TH neurons could be activated by CNO. Briefly, LC TH-hM3Dq mice were injected i. p. with CNO (1 mg/kg) or saline and then killed 90 min later for c-Fos immunohistochemistry.

In the induction and emergence test, mice were placed in a cylindrical chamber filled with 1.2% isoflurane in oxygen at a flow rate of 1 L/min. The time interval from isoflurane exposure to loss of righting reflex (LORR) was recorded as induction time. The cylindrical chamber was gently rotated by 90° every 15 s until the mouse on its back and loss the ability to right itself (Luo et al., 2018; Wang et al., 2019). In addition, the time interval between mice removed from the chamber and return righting reflex (RORR) with all four paws on the ground was recorded as emergence time. LORR and RORR are well-established surrogate measure of rodent animals for determining the onset and recovery of general anesthesia, respectively (Franks, 2008). The investigator was blinded to the allocated groups. In electroencephalogram (EEG) test, after 1 h of CNO (1 mg/kg, i. p.) or saline injection, mice were anesthetized (1.2% or 0.8% isoflurane), and EEG were recorded continuously for 30 min (Figure 3B). The burst suppression ratio (BSR) was calculated as the percentage of EEG suppression in the 2 min interval before cessation of 1.2% isoflurane. Power spectrum analysis was conducted on the 2 min interval before cessation of isoflurane and the 2 min interval after cessation of isoflurane.

#### c-Fos Expression in the PVT After Chemogenetic Activation of LC TH Neurons

In order to weaken the unfamiliar stimuli and new environment stimuli, mice were placed into the cylindrical chamber individually for at least 1 h with 100% oxygen (1 L/min) flowing starting at least 3 days before the experiment. On the day of the experiment, CNO (1 mg/kg) or saline were administered i. p. in hM3Dq mice 1 h before isoflurane application. After 1 h conditioning, 1.2% isoflurane was administered to the chamber for 30 min. At the end of isoflurane anesthesia, mice were killed for c-Fos immunohistochemical experiments. There was no other operation for mice during this time, and all experiments were conducted in the light phase.

#### Optogenetic Activation Experiments

For optogenetic activation experiments, AAV-EF1a-DIO-ChR2(H134R)-EYFP or AAV-EF1a-DIO-EYFP was injected bilaterally into the LC of TH-Cre mice. After at least 3 w, optical fibers were implanted above the PVT. During test, optical stimulation was performed with blue light (473 nm) from a laser (Shanghai Dream Lasers Technology Co., Ltd., China). The power of the 473 nm laser at the tip of the fiber was 12–15 mW measured using an optical power meter (PM100D, Thorlabs, United States). The 473 nm blue light pulse trains (10 Hz, 10 ms duration) were applied to excite the TH:LC-PVT projections (Ren et al., 2018). In the optogenetic experiments, EEG were recorded continuously for 40 min. Optical manipulation (10 Hz, 1 min) was applied after constant 30 min 1.2% or 0.8% isoflurane administration (Figure 5B). BSR and power spectrum analysis were calculated from 1 min interval before and during the optical stimulation. In the induction test (Figure 5I), optical stimulation was administered at the start of 1.2% isoflurane delivery until mice lost righting reflex in the cylindrical chamber. In the emergence test (Figure 5J), mice were anesthetized for 30 min, once the isoflurane delivery turned off, sustained optical stimulation was delivered until the mice returned righting reflex.

#### Chemogenetic Inhibition Experiments

For chemogenetic inhibition experiments, AAV-hSyn-DIO-hM4Di-mCherry or AAV-EF1a-DIO-EYFP was injected bilaterally into the LC of TH-Cre mice. After at least 3 w, guide cannulae were implanted above the PVT. CNO (5 μM, 500 nl) or the same volume saline was locally infused into PVT at a rate of 250 nl/min by a microinjection pump (KD Scientific, United States) in each mouse 20 min before anesthesia. After injection, the infusion cannula was left for 2 min to allow for infusion. Each mouse was administered with CNO or saline separated by at least 3 days washout period. The experimental protocol was shown in Figure 6B. Induction time and emergence time were recorded as described above.

### Electroencephalogram Analysis

EEG signals were amplified and collected by a RM-6240EC device (Chengdu Instrument Factory, China) at a sampling rate of 800 Hz. The signals were filtered between 0.1 and 100 Hz. The total power spectrum, BSR, and power distribution in each frequency band were completed by MATLAB 2016a (The MathWorks, United States). The BSR was calculated by using previously established methods (Vijn and Sneyd, 1998; Vazey and Aston-Jones, 2014). Power spectrum analysis was conducted as previously described (Wang et al., 2019). Delta, theta, alpha, beta, gamma and total spectral powers were calculated using the frequency bands 1–4 Hz, 4–8 Hz, 8–15 Hz, 15–30 Hz and 30–50 Hz, respectively. Relative powers were calculated by dividing the averaged signal power across each band’s frequency range by the total power of 1–50 Hz.

### Immunohistochemistry

Mice were deeply anesthetized and transcardially perfused with ice-cold 0.9% saline, followed by 4% paraformaldehyde. The brains were harvested and post-fixed overnight at 4°C. Brain was continuously sectioned into 40 μm coronal slices using a vibratome (Leica VT1000S, Leica Biosystems, Germany). Brain sections were incubated in 1 × PBS solution containing 0.3% Triton X-100 and 10% normal goat serum (Bosterbio, United States). Sections were incubated with primary antibody diluted in PBS plus 10% normal goat serum overnight at 4°C for 24 or 48 h. Incubated slices were then washed by PBS three times (10 min each), then incubated for 90 min with secondary antibody in PBS, and subsequently washed three times in PBS (10 min each) at room temperature. Primary antibodies used: rabbit polyclonal anti-c-Fos (1:3000, Synaptic Systems Cat# 226003, RRID:AB_2231974, Germany) and mouse monoclonal anti-TH (1:1000, Millipore Cat# MAB318, RRID:AB_2201528, United States). Secondary antibodies were Alexa Fluor 488 goat anti-rabbit (Thermo Fisher Scientific Cat# A-11008, RRID:AB_143165, United States), Alexa Fluor 488 goat anti-mouse (Thermo Fisher Scientific Cat# A-11029, RRID:AB_2534088, United States), Alexa Fluor 594 goat anti-rabbit (Thermo Fisher Scientific Cat# A-11012, RRID:AB_2534079, United States), Alexa Fluor 594 goat anti-mouse (Thermo Fisher Scientific Cat# A-11032, RRID:AB_2534091, United States) and Alexa Flour 647 donkey anti-mouse (Thermo Fisher Scientific Cat# A-31571, RRID:AB_162542, United States). Secondary antibodies were diluted with 1:600 upon use. After staining DAPI (5 μg/ml, Cat#: 10236276001, Roche, Switzerland) and washing three times with PBS, sections were mounted on glass microscope slides, dried and covered with mounting media.

### Imaging and Quantification

Images were captured using a laser confocal fluorescent microscope (Lecia sp8, Germany). Quantification of c-Fos within the PVT was performed as described previously (Ren et al., 2018; Ao et al., 2020). Briefly, the number of c-Fos positive neurons in the PVT was counted at alternate sections from approximately Bregma −0.94 and to −1.46 mm (five sections per mouse) along the rostral-caudal axis. The investigator blinded to the group information manually counted the c-Fos-positive cells in a ROI (a square area with a side length of 500 µm) located within PVT in each section using ImageJ (National Institutes of Health) software. Colocalization of c-Fos and TH immunofluorescence in the LC was quantified on adjacent sections from approximately bregma −5.3 to −5.7 mm (five sections per mouse) using ImageJ. The location of PVT or LC was based on mouse brain atlas (Paxinos and Franklin, 2004). If the viral transfection was poor, or the optical fiber and guide cannula site was incorrect, the mouse’s associated data was excluded.

### Statistical Analysis

All statistical analyses were performed using the Graphpad Prism 7.0. All data were presented as mean ± SEM. Sample sizes were determined based on previous publications on general anesthesia regulation using chemogenetic and optogenetic approaches (Taylor et al., 2016; Zhou et al., 2018; Yin et al., 2019; Wang et al., 2020). We first performed the normality test on each dataset using the Shapiro-Wilk test. Otherwise, non-parametric tests were used. Two-tailed paired and unpaired t tests, one-way ANOVA and two-way ANOVA followed by the Tukey’s multiple comparisons test were used. Statistical significance was set at *p* < 0.05.

## Results

### The LC TH Neurons Are Active During Passive Emergence From Isoflurane Anesthesia

Given that LC plays an important role in changing the arousal level (Carter et al., 2010; Benarroch, 2018), it is possible that LC participates in emergence from anesthesia. Thus, we subjected the wild type mice to 100% oxygen exposure alone (Oxy group), 1.2% isoflurane anesthesia (ISO group) and 1.2% isoflurane anesthesia with emergence (EM group) followed by examining c-Fos expression in LC. The experimental protocol was shown in Figure 1A. We calculated the percentage of c-Fos + TH/TH, and found that the percentage in EM group (71.5 ± 3.6%, *n* = 4) was significantly higher than that in the ISO group (20.7 ± 1.5%, *n* = 3) and Oxy group (40.1 ± 6.1%, *n* = 3) (Figures 1B–E) [F (2, 7) = 40.82, *p* = 0.0001]. The massive increase in c-Fos activity in the EM group suggests that the LC TH neurons may participate in passive emergence from isoflurane anesthesia.

### LC TH Neurons Activation Facilitates Emergence From Anesthesia

**FIGURE 2:**
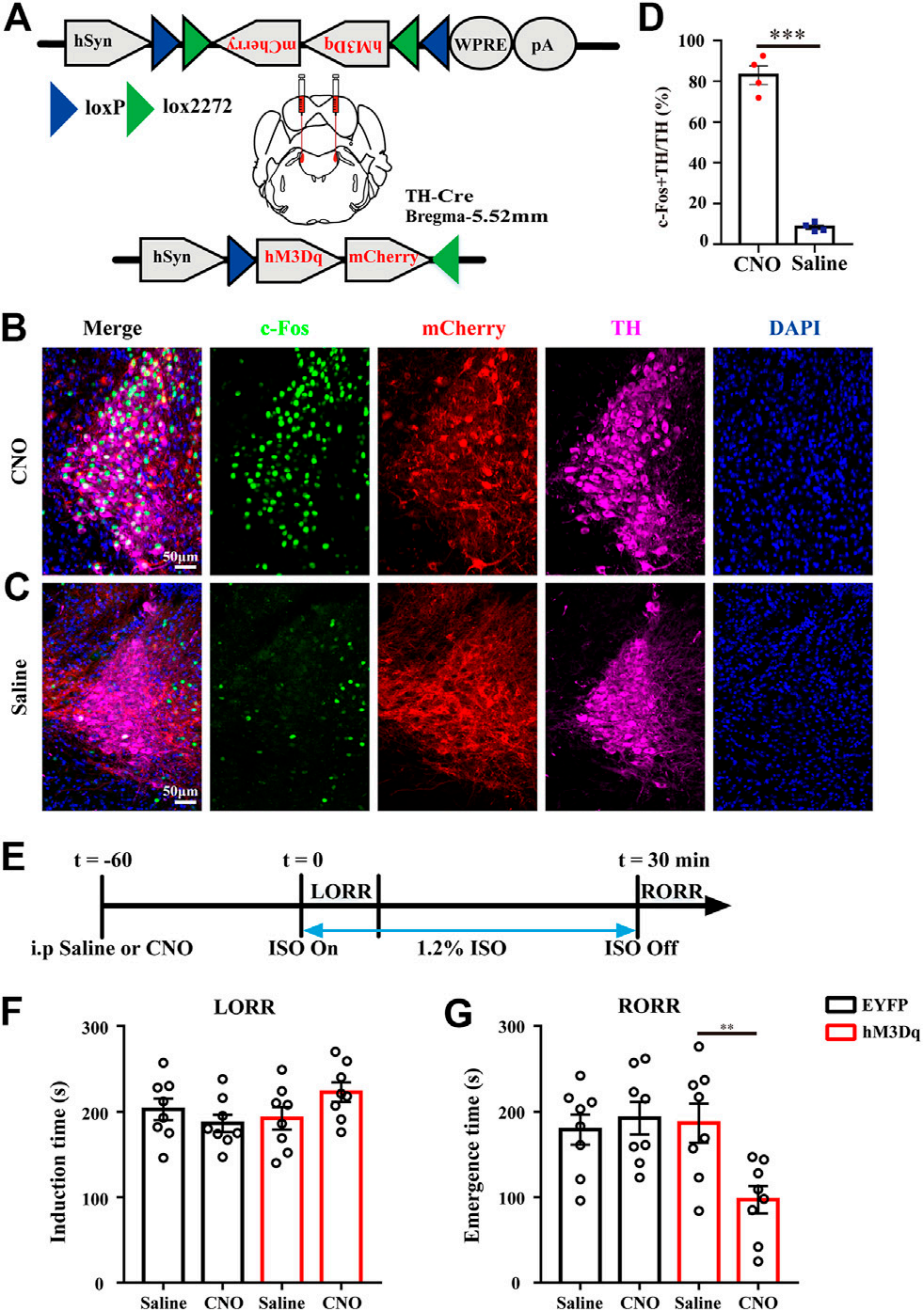
LC TH neurons activation facilitates emergence from anesthesia. **(A)**Schematic diagram showing the injection target of LC in TH-Cre mice and representation of hM3Dq vectors injected under control of the hSyn promoter. **(B,C)** Representative images showing the co-localization of c-Fos (green), TH (purple) and mCherry (red) in LC of hM3Dq mice treated with CNO **(B)** or saline **(C)**. **(D)** The percentage of c-Fos + TH/TH in the LC of hM3Dq mice injected i. p. with CNO or saline (*n* = 4 in each group, the two-tailed unpaired t-test, ***p < 0.001). **(E)**Timeline for measuring induction and emergence time in hM3Dq mice injected i. p. with CNO or saline. **(F)** Induction time with exposure to 1.2% isoflurane after injected i. p. with saline or CNO of EYFP (black) and hM3Dq (red) mice, respectively (*n* = 8 in each group, two-way RM ANOVA with Tukey’s multiple comparisons test). **(G)** Emergence time with exposure to 1.2% isoflurane after treatment with saline or CNO of EYFP (black) and hM3Dq (red) mice, respectively (*n* = 8 in each group, two-way RM ANOVA with Tukey’s multiple comparisons test, **p < 0.01). Data shown as means ± SEM.

The higher activity in LC TH neurons during anesthesia emergence suggests that the LC may be important for recovery from anesthesia. To express the DREADD receptor hM3Dq in LC TH neurons, AAV-hSyn-DIO-hM3Dq-mCherry were stereotaxically injected in LC of TH-cre mice. This AAV construct contains a FLEX switch, which includes loxP and lox2272, to ensure stable expression of hM3Dq in Cre-expressing cells (Figure 2A). To confirm hM3Dq receptor excitation lead to increased TH neuronal activity *in vivo*, the LC TH-hM3Dq mice were injected i. p. with CNO (1 mg/kg) or saline and killed 90 min later for c-Fos immunohistochemistry (Figures 2B,C). We calculated the percentage of c-Fos + TH/TH and found that CNO increased the percentage in the LC, it was 83.0 ± 4.6% in the CNO group (*n* = 4) and 8.5 ± 1.1% in the saline group (*n* = 4)[ t (6) = 15.79, *p* = 0.0003] (Figure 2D).

To test whether activation of LC TH neurons could modulate the speed of induction and emergence from anesthesia, 60 min after CNO or saline injection, mice were subjected to the induction and emergence test. Figure 2E showed the experimental protocol. We set EYFP infected mice treated with either saline or CNO and hM3Dq infected mice treated with saline as control groups. Compared with control groups, activation of LC TH neurons did not obviously change induction time by two-way ANOVA multiple comparisons [F (1, 7) = 3.884, *p* = 0.0894] (Figure 2F). However, activation of LC TH neurons shortened the emergence time (Figure 2G). The hM3Dq mice injected with CNO (97.3 ± 15.9 s, *n* = 8) showed a significant decrease in emergence time relative to the hM3Dq-saline group (186.8 ± 22.8 s, *n* = 8), the EYFP-CNO group (192.5 ± 19.0 s, *n* = 8) and the EYFP-saline group (179.1 ± 17.7 s, *n* = 8) [F (1, 7) = 17.54, *p* = 0.004] (Figure 2G). Taken together, these results suggest that LC TH neurons activation facilitates emergence from isoflurane anesthesia.

### LC TH Neurons Activation Drives Cortical Arousal

**FIGURE 3:**
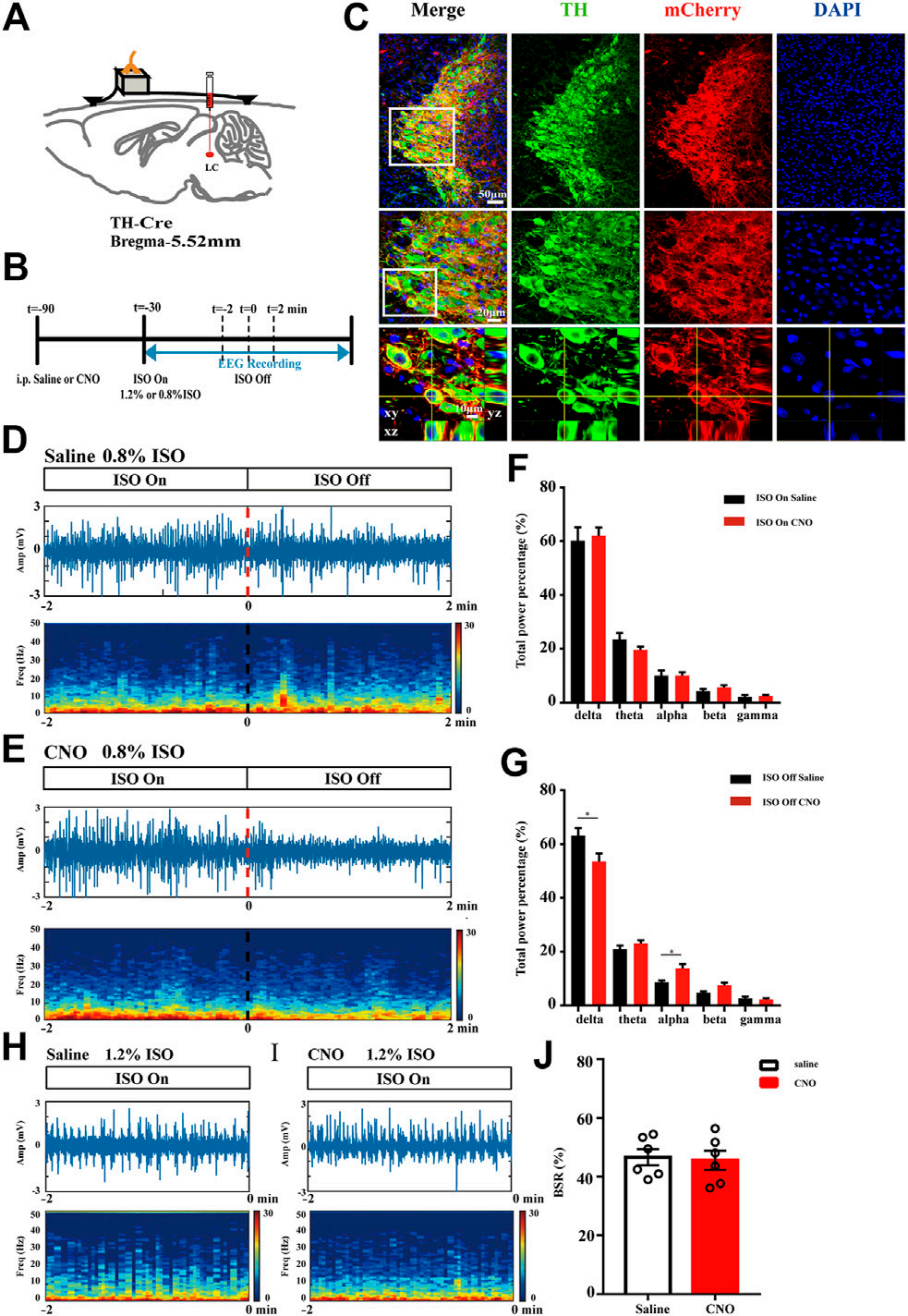
LC TH neurons activation drives cortical arousal. **(A)** Schematic diagram showing bilateral virus injection and EEG recording. **(B)** Timeline for EEG recording during isoflurane anesthesia and emergence in hM3Dq mice injected i. p. with CNO or saline. **(C)** Specific expression of hM3Dq-mCherry (red) in LC TH (green) neurons. Insets were amplified in below. **(D,E)** A representative raw EEG trace (top) and the corresponding power spectrogram (bottom) during 0.8% isoflurane anesthesia and emergence period in hM3Dq-saline **(D)** and hM3Dq-CNO **(E)** mice. 0 on the time axis indicates the cession of isoflurane. **(F)** Power spectrum analysis was conducted on 2 min window before cession of isoflurane in hM3Dq-saline and hM3Dq-CNO mice (*n* = 6 in each group, the two-tailed unpaired t-test). **(G)** In the emergence period, power spectrum analysis was conducted from 0.8% isoflurane inhalation stop to 2 min window (*n* = 6 in each group, the two-tailed unpaired t-test, *p < 0.05). **(H,I)** A representative raw EEG trace (top) and the corresponding power spectrogram (bottom) during 1.2% isoflurane anesthesia in hM3Dq-saline **(H)** and hM3Dq-CNO **(I)** mice. **(J)** In the anesthesia period, BSR was calculated at 2 min window before cession of isoflurane in hM3Dq-saline and hM3Dq-CNO mice (*n* = 6 in each group, the two-tailed unpaired t-test). Data shown as means ± SEM.

Having performed behavior tests to reveal facilitated anesthesia recovery with LC TH neurons excitation, we next evaluated the impact of LC TH neurons activation on the brain activity under anesthesia by EEG recording. Figure 3A showed schematic of bilateral LC injection and EEG recording. Timeline for EEG recording during isoflurane anesthesia and emergence was shown in Figure 3B. Specific expression of hM3Dq-mCherry in LC TH neurons were shown in Figure 3C. Power spectrum analysis showed that the percent distributions of the five frequency bands were similar between the CNO group (*n* = 6) and the saline group (*n* = 6) while the mice were exposed to 0.8% isoflurane (Figures 3D–F). However, upon switching off the 0.8% isoflurane, the CNO group showed a significant decrease of delta power [t (10) = 2.325, *p* = 0.0424] and increase of alpha power [*U* = 3.000, *p* = 0.0152] in the first 2 min (Figures 3D,E,G). However, during the 1.2% anesthesia period, there was no significant difference in BSR between the CNO group (*n* = 6) and the saline group (*n* = 6) [t (10) = 0.2392, *p* = 0.8157] (Figures 3H–J). Therefore, the results demonstrate that the LC TH neurons excitation promotes cortical arousal during anesthesia emergence.

### LC TH Neurons Activation Enhances Neuronal Activity in Paraventricular Thalamus

**FIGURE 4:**
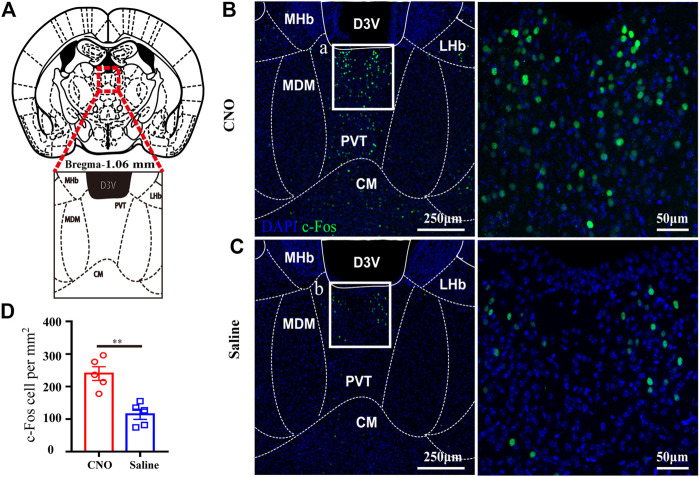
LC TH neurons activation enhances neuronal activity in PVT. **(A)** Schematic diagram showing the location of the PVT and local structures in mouse. **(B,C)** Representative photomicrographs of c-Fos expression in the PVT in hM3Dq- CNO and hM3Dq-saline mice. Insets were amplified on the right. **(D)** Quantification of c-Fos positive nuclei in the PVT of hM3Dq-saline and hM3Dq-CNO mice (*n* = 5 in each group, two-tailed unpaired t-test, **p < 0.01). Data shown as means ± SEM.

Given that the PVT is a central node for wakefulness and it heavily receives projections from the LC (Beas et al., 2018; Ren et al., 2018), we evaluated PVT neurons activity after LC TH neuronal excitation in hM3Dq mice. Figure 4A showed the location of the PVT and local structures. The representative photomicrographs of c-Fos expression in the PVT of hM3Dq mice treated with CNO or saline were shown in Figures 4B,C. After CNO or saline injection in LC TH-hM3Dq mice respectively, the number of c-Fos positive nuclei in PVT was 239.8 ± 21.3% in the CNO group (*n* = 4) and 114.7 ± 15.7% in the saline group (*n* = 4) [t (10) = 4.733, *p* = 0.0019] (Figure 4D), suggesting that LC TH neurons excitation enhances the PVT activity, and the PVT may be a critical target of LC to drive emergence from anesthesia.

### Optogenetic Activation of TH:LC-PVT Terminals Promotes Arousal From Isoflurane Anesthesia

**FIGURE 5:**
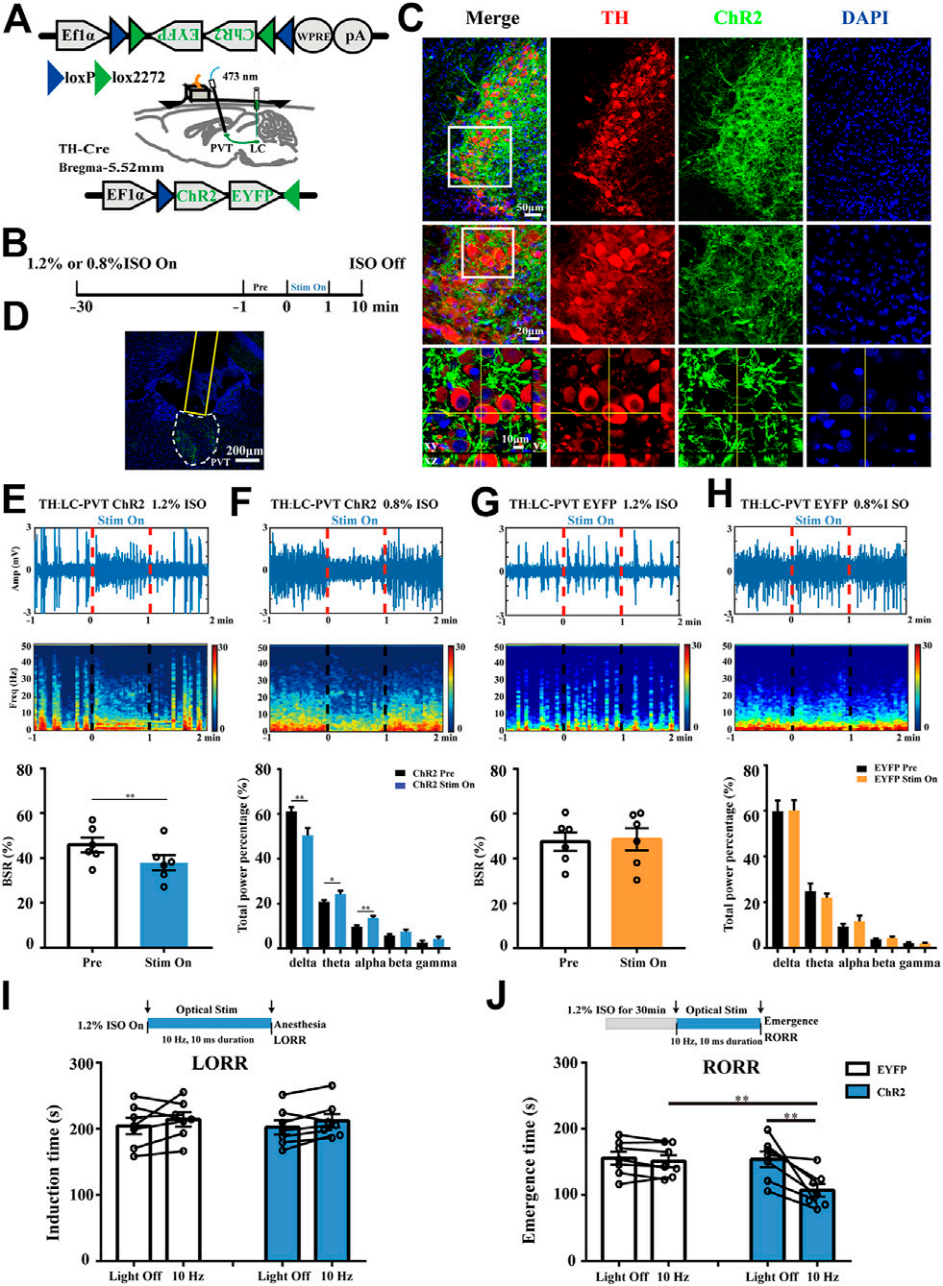
Optogenetic activation of TH:LC-PVT terminals promotes arousal from isoflurane anesthesia. **(A)** Schematic of bilateral LC injection, optic fiber implantation and EEG recording. **(B)** Experimental protocol for optical stimulation and EEG recording during isoflurane anesthesia. After 30 min inhalation of isoflurane, the mice were optically stimulated for 1 min (stim on). **(C)** Specific expression of ChR2-EYFP (green) in LC TH (red) neurons. Insets were amplified in below **(D)** Representative ChR2-EYFP terminals and the location of optical fiber in the PVT. **(E,F)** A representative raw EEG trace (top) and the corresponding power spectrum (middle) at 1 min before, 1 min during and 1 min after optical stimulation in ChR2 mouse during 1.2% **(E)** or 0.8% **(F)** isoflurane anesthesia. BSR was calculated at 1 min before and 1 min during optical stimulation in ChR2 mice (bottom, *n* = 6, two-tailed paired t-test, **p < 0.01). EEG power percentage of different frequency bands at 1 min before (black) and 1 min during (blue) optical stimulation in ChR2 mice (bottom, *n* = 6, the two-tailed paired t-test, *p < 0.05, **p < 0.01). **(G,H)** A representative raw EEG trace **(top)** and corresponding power spectrum **(middle)** at 1 min before, 1 min during and 1 min after optical stimulation in EYFP control mouse during 1.2% **(G)** or 0.8% **(H)** isoflurane anesthesia. BSR was calculated at 1 min before and 1 min during optical stimulation in control mouse (bottom, *n* = 6, the two-tailed paired t-test). EEG power percentage of different frequency bands at 1 min before (black) and 1 min during (blue) optical stimulation in control mice (bottom, *n* = 6, two-tailed paired t-test) **(I)** Experimental protocol for optical stimulation during 1.2% isoflurane anesthesia induction **(top)**. Effects of activating TH:LC-PVT terminals on the induction time in EYFP (control) and ChR2 mice (bottom, *n* = 7 in each group, two-tailed paired t-test). **(J)** Experimental protocol for optical stimulation during 1.2% isoflurane anesthesia emergence **(top)**. Effects of activating TH:LC-PVT terminals on the emergence time in EYFP (control) and ChR2 mice (bottom, *n* = 7 in each group, two-tailed paired t-test or two-tailed unpaired t-test, **p < 0.01). Data shown as means ± SEM.

To test whether the TH:LC-PVT projections could promote arousal from general anesthesia, we placed optical fibers above the PVT of LC TH-ChR2-EYFP mice to stimulate the LC TH terminals (Figure 5A). The timeline for optogenetic activation and EEG recording during isoflurane anesthesia was shown in Figure 5B. After 3–4 w virus expression, specific expression of ChR2-EYFP were observed in LC TH neurons (Figure 5C). In addition, dense EYFP fibers were observed in the PVT (Figure 5D). Optically stimulating LC TH fibers in the PVT reduced BSR from 45.8 ± 3.3% to 37.9 ± 3.4% during 1.2% isoflurane anaesthesia in ChR2 mice (*n* = 6) [t (5) = 5.305, *p* = 0.0032] (Figure 5E), but not in control mice (*n* = 6) [t (11) = 0.7344, *p* = 0.4957] (Figure 5G). Besides, optogenetic activation of LC TH fibers produced a decrease in delta power (*n* = 6) [t (5) = 4.668, *p* = 0.0055], whereas the power in theta (*n* = 6) [t (5) = 2.719, *p* = 0.0418] and alpha (*n* = 6) [t (5) = 5.098, *p* = 0.0038] was statistically increased. However, there was no difference in the EEG power spectrum in control mice with stimulation (*n* = 6) (Figure 5H). In addition, optical excitation of LC TH fibers in the PVT altered EEG activity in the ChR2 mice from an anesthesia state to an awake-like state and returned to an anesthesia state after cessation of stimulation (Figures 5E,F).

To further investigate whether activation of TH:LC-PVT projections were sufficient to influence induction and emergence from isoflurane anesthesia, we designed the experiment as shown in Figures 5I,J. Optical stimulation of LC TH terminals in the PVT did not significantly alter the induction time under 1.2% isoflurane in ChR2 (*n* = 7) [t (6) = 1.197, *p* = 0.2763] or control mice (*n* = 7) [t (6) = 1.211, *p* = 0.2714] (Figure 5I). Notably, photostimulation of TH:LC-PVT projections in ChR2 mice facilitated emergence from isoflurane anaesthesia. The emergence time was shortened from 153.6 ± 11.9 s to 106.9 ± 9.9 s in ChR2 mice (*n* = 7) [t (6) = 3.712, *p* = 0.0099], but not in control mice (*n* = 7) [t (6) = 1.465, *p* = 0.1933] (Figure 5J). Taken together, these findings indicate that TH:LC-PVT projections are sufficient for anesthesia emergence.

### Chemogenetic Inhibition of TH:LC-PVT Terminals Delays Emergence From Anesthesia

**FIGURE 6:**
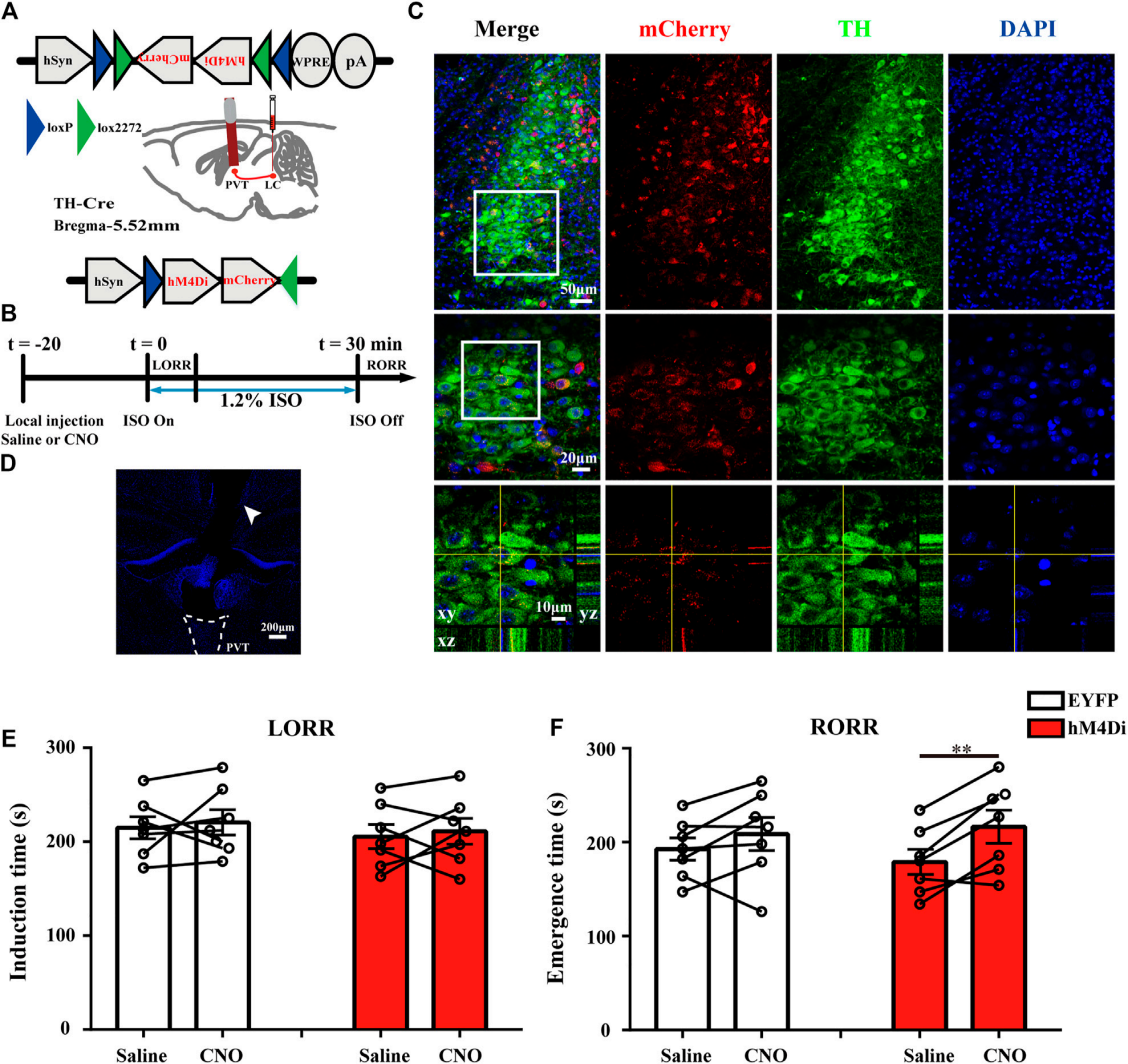
Chemogenetic inhibition of TH: LC-PVT terminals delays emergence from isoflurane anesthesia. **(A)** Schematic of bilateral LC injection and guide cannula implantation. **(B)** Experimental protocol for chemogenetic inhibition of LC TH terminals in the PVT during 1.2% isoflurane anesthesia. **(C)** Specific expression of hM4Di-mCherry (red) in LC TH (green) neurons. Insets were amplified in below. **(D)** DAPI stained coronal brain slice showing the track of the guide cannula (white arrow head) above the PVT. **(E)** Inhibition of TH:LC-PVT terminals showed no significant changes of induction time in hM4Di mice (*n* = 7 in each group, two-tailed paired t-test). **(F)** Inhibition of TH:LC-PVT terminals significantly delayed emergence time in hM4Di mice (*n* = 7 in each group, two-tailed paired t-test, **p < 0.01). Data shown as means ± SEM.

To verify the necessity of this specific pathway in regulating the arousal from anesthesia, we placed cannulae above the PVT of LC TH-hM4Di-mCherry mice to suppress the LC TH terminals by local CNO microinjection (Figure 6A). The timeline for chemogenetic inhibition and behavioral tests was shown in Figure 6B. After 3–4 w virus expression, specific expression of hM4Di-mCherry were observed in LC TH neurons (Figure 6C). The positions of the cannulae were verified (Figure 6D). Application of CNO or saline in hM4Di mice (*n* = 7) [t (6) = 0.4561, *p* = 0.6643] showed no significant difference in induction time, as well as in EYFP mice (*n* = 7) [t (6) = 0.4375, *p* = 0.6771] (Figure 6E). However, chemogenetic inhibition of TH:LC-PVT projections significantly prolonged emergence time in hM4Di mice treated with CNO (216.4 ± 17.7 s) (*n* = 7), which compared with same mice treated with saline (178.9 ± 13.3s) (*n* = 7) [t (6) = 4.205, *p* = 0.0057] (Figure 6F), but not in EYFP mice (*n* = 7) [t (6) = 1.436, *p* = 0.2010] (Figure 6F). Thus, these findings indicate that TH:LC-PVT projections are vital for anesthesia emergence.

## Discussion

In the current study, we identified that LC TH neurons were active during passive emergence from isoflurane anesthesia and chemogenetic activation of LC TH neurons facilitated emergence from anesthesia. In addition, excitation of LC TH neurons promoted cortical arousal and enhanced PVT activity. Furthermore, optogenetic activation of the TH:LC-PVT projections facilitated arousal, whereas selective inhibition of this circuit delayed arousal from isoflurane anesthesia. Our findings indicate that TH:LC-PVT pathway is involved in emergence from anesthesia.

It has been demonstrated that brain regions that regulate arousal become active during anesthesia recovery process (Muindi et al., 2016; Luo et al., 2018; Luo et al., 2020). Additionally, the histaminergic (Luo and Leung, 2009), cholinergic (Irmak and de Lecea, 2014), noradrenergic (Berridge et al., 2012; Fu et al., 2017), dopaminergic (Eban-Rothschild et al., 2016; Taylor et al., 2016), and orexinergic pathways (Sakurai, 2007) have been implicated in linking sleep-wake regulation and the emergence from GA. Our c-Fos immunostaining data reveals that LC TH neurons are active during emergence from isoflurane anesthesia. A previous study has documented that LC noradrenergic neurons promote the emergence from general anesthesia (Vazey and Aston-Jones, 2014). Thus, our study extends previous findings on the role of LC noradrenergic system in anesthetic arousal. Specifically, activation of LC TH neurons drove cortical arousal, accelerated behavioral emergence from isoflurane anesthesia and enhanced the PVT activity. Thus, the results indicate that LC TH neurons probably serve as a key element to induce the recovery from GA and this effect may be mediated by activation of the PVT. It is worth noting that LC TH neurons excitation shortened anesthetic emergence but had no singnificant impact on anesthetic induction. The present work adds to the growing body of evidence that the induction and emergence processes are not merely the mirror image of each other, and emergence is an active process characterized by a distinct neurobiology (Kelz et al., 2008; Tarnal et al., 2016).

The anesthesia emergence is of critical importance (Hight et al., 2014). For example, the strategies for safer and active reversal of anesthesia can be designed to bring consciousness recovery as quickly as possible. The PVT in the thalamus’s paramedian region is essential for wakefulness (Colavito et al., 2015; Ren et al., 2018; Shao et al., 2019). Furthermore, the PVT receives profuse innervation from LC TH neurons, but receives little innervation by midbrain dopaminergic neurons (Li et al., 2014; Beas et al., 2018). In the present study, optogenetic activation of the TH:LC-PVT projections drove cortical arousal and facilitated emergence, whereas inhibition of TH:LC-PVT projections prolonged emergence. The measures of increased cortical arousal, including a reduction in BSR, decreases in delta power, and increases in theta power and alpha power, were seen after optical stimulation of TH:LC-PVT projections. Thus, LC-PVT projections have a potential use to facilitate the emergence from anesthesia. In this study, we have confirmed the role of the TH:LC-PVT projections in facilitating emergence from anesthesia, however the downstream of the TH:LC-PVT pathway is not clear. The nucleus accumbens (NAc) and bed nucleus of the stria terminalis (BNST) are innervated by the PVT and play roles in sleep-wake control (Ren et al., 2018) (Hua et al., 2018), thus the TH:LC-PVT projections may activate the NAc or BNST to facilitate the emergence from anesthesia (Kirouac, 2015).

Despite the evidence supporting that LC is a noradrenergic center, accumulating data suggest that LC neurons’ terminals may co-release dopamine (DA) and norepinephrine (NE) (Devoto et al., 2005; Kempadoo et al., 2016; Takeuchi et al., 2016; Beas et al., 2018). DA and NE have been studied as two different systems, however, they have many similarities such as shared biosynthetic pathway, co-release from TH terminals, innervation of similar regions, and shared intracellular signaling pathways (Ranjbar-Slamloo and Fazlali, 2019). Both NE and DA are well-known to contribute to arousal (Monti and Monti, 2007; Fu et al., 2017). Accordingly, it has been shown that methylphenidate, which is a potent inhibitor of DA and NE transporters, induces the active emergence from anesthesia, indicating that both DA and NE may have synergistic effects (Solt et al., 2011; Chemali et al., 2012).

The current study has several limitations. First, we used only male mice in the experiment. Whether this circuit also promotes anesthesia arousal in female mice remains unclear. Second, we did not measure body fat, liver function and metabolites. The slower emergence in patients under general anesthesia may be due to obesity (Casati and Putzu, 2005). However, we used mice in the normal weight range, so the difference of emergence time was less likely to be resulted from obesity in our study. Nevertheless, the obesity-induced arousal rate change needs further exploration. In addition, volatile anesthetics may be metabolized to reactive and potentially toxic intermediates (Martin, 2005). These metabolites may lead to hepatotoxicity (Singhal et al., 2010; Rajan et al., 2019). In turn, abnormal liver function and metabolism may lead to slow metabolism of anesthetics. Thus, liver function and metabolic abnormalities need to be monitored in the future study to rule out potential factors affecting arousal rate.

## Conclusion

In summary, stimulation of the TH:LC-PVT terminals is sufficient to accelerate the transition from general anesthesia to an arousal state. A clear understanding of the neural basis is fundamental to research in clinical and basic neuroscience disciplines and anesthesia.

## Data Availability

The original contributions presented in the study are included in the Supplementary Material, further inquiries can be directed to the corresponding author.

## Abbreviations

LC, Locus coeruleus; AVV, adeno-associated virus; EEG, electroencephalogram; TH, tyrosine hydroxylase; Cre, Cre recombinase; GA, general anesthesia; DA, dopamine; NE, norepinephrine; LORR, loss of the righting reflex; RORR, return of the righting reflex; CNO; clozapine N-oxide, BSR, burst suppression ratio; ChR2; channelrhodopsin-2; EYFP, enhanced yellow fluorescent protein; NAc, nucleus accumbens; BNST, bed nucleus of the stria terminalis.
