# Supplementary material for: Locus Coeruleus to Paraventricular Thalamus Projections Facilitate Emergence From Isoflurane Anesthesia in Mice
Source: Front Pharmacol. 2021 Apr 27;12:643172. doi: 10.3389/fphar.2021.643172 (PMC8111010; doi:10.3389/fphar.2021.643172)
Supplement: Supplementary file 1 [file DataSheet1.PDF]

Figure number	N NUMBER	Normality test	Statistic method	F/t value	DESCRIPTIVE STATS<br>Error bars are mean+/-<br>SEM<br>*p* value
Figure 1 E	*n* = 3 for ISO mice<br>, *n* = 3 for Oxy mice and *n* = 4 for EM mice	passed	one-way ANOVA with post hoc Bonferroni	F (2, 7) = 40.82	ISO (20.7 ± 1.5%)<br>OXY (40.1 ± 6.1%)<br>EM (71.5 ± 3.6%)<br>ISO vs. OXY $p < 0.05$<br>ISO vs. EM $p < 0.0001$<br>OXY vs. EM $p < 0.001$
Figure2 D	*n* = 4 for each group	passed	Unpaired t test (two-tailed)	t(6) = 15.79	saline (8.5 ± 1.1%)<br>CNO (83.0 ± 4.6)<br>saline vs. CNO<br>$p < 0.001$
Figure 2 F	*n* = 8 for each group	passed	Two-way RM ANOVA with Tukey's multiple comparisons test	F (1, 7) = 3.884	EYPF-saline (202.9 ±12.62)<br>EYPF-CNO (186.5 ± 10.23)<br>hM3Dq-saline (192.3 ±13.18)<br>hM3Dq-CNO (222.8 ± 11.43)<br>$p > 0.05$
Figure 2 G	*n* = 8 for each group	passed	Two-way RM ANOVA with Tukey's multiple comparisons test	F (1, 7) = 17.75	EYPF-saline (179.1 ±17.69)<br>EYPF-CNO (192.5 ±18.96)<br>hM3Dq-saline (186.8 ± 22.83)<br>hM3Dq-CNO (97.25 ± 15.91)<br>saline :hM3dq vs. CNO :hM3dq<br>$p < 0.01$
Figure 3 F	*n* = 6 for each group	passed	Unpaired t test (two-	t (10) = 0.3372<br>t (10) = 1.45	Saline delta (60.18 ± 4.923%) vs

			tailed)	t (10) = 0.03652<br>t (10) = 1.311<br>t (10) = 0.4964	CNO delta<br>(62.11 ± 2.94 %)<br>$p > 0.05$<br>Saline theta (23.42 ± 2.42%) vs CNO theta<br>(19.55 ± 1.13 %)<br>$p > 0.05$<br>Saline alpha (10 ± 1.94%) vs CNO alpha<br>(10.09 ± 1.16%)<br>$p < 0.05$<br>Saline beta (4.273 ± 0.80%)<br>vs CNO beta<br>(5.736 ± 0.78 %)<br>$p > 0.05$<br>Saline gamma<br>(2.127 ± 0.68%)<br>vs CNO gamma<br>(2.514 ± 0.38 %)<br>$p > 0.05$
Figure 3 G	$n = 6$ for each group	passed	Unpaired t test (two-tailed)	t (10) = 2.325<br>t (10) = 1.204<br>t (10) = 2.2<br>t (10) = 0.588	Saline delta (63.09 ± 2.87%) vs CNO delta<br>(53.6 ± 2.91%)<br>$p < 0.05$<br>Saline theta (20.91 ± 1.31%) vs CNO theta<br>(23.05 ± 1.2 %)<br>$p > 0.05$<br>Saline alpha (8.62 ± 0.69%) vs CNO alpha<br>(13.75 ±1.61%)<br>$p < 0.05$<br>Saline beta (4.72 ± 0.54%)<br>vs CNO beta<br>(7.42 ± 1.10 %)<br>$p > 0.05$<br>Saline gamma

					(2.65 ± 0.67%) vs CNO gamma (2.18 ± 0.45 %) $p > 0.05$
Figure 3 H	*n* = 6 for each group	passed	Unpaired t test (two-tailed)	t (10)=0.2392	1.2% ISO On BSR saline (46.61 ± 2.73 %) 1.2% ISO On BSR CNO (45.59 ± 3.25 %) $p > 0.05$
Figure 4 D	*n* = 5 for each group	passed	Unpaired t test (two-tailed)	t (8) = 4.733	saline (114.7 ± 15.71) CNO (239.8 ± 21.26) $p < 0.01$
Figure 5 E	*n* = 6 for each group	passed	paired t test (two-tailed)	t (5) = 5.305	Pre (45.8 ± 3.3%) vs Stim on (37.9 ± 3.4%) $p < 0.01$
Figure 5 F	*n* = 6 for each group	passed	paired t test (two-tailed)	t (5) = 4.668<br>t (5) = 5.098<br>t (5) = 2.719<br>t (5) = 2.052<br>t (5) = 1.541	Pre delta (61.1 ± 1.95%) vs Post delta (50.41 ± 3.30 %) $p < 0.01$<br>Pre theta (20.83 ±0.72%) vs Post theta (24.33 ± 1.41 %) $p < 0.01$<br>Pre alpha (9.63 ± 0.69%) vs Post alpha (13.59 ± 0.99 %) $p < 0.05$<br>Pre beta (5.72 ±0.65%) vs Post beta (7.51 ± 1.01 %) $p > 0.05$<br>Pre gamma (2.72 ± 0.81%) vs Post gamma (4.17 ± 4.17 %)

					$p > 0.05$
Figure 5 G	$n = 6$ for each group	passed	paired t test (two-tailed)	t (5) = 0.7344	Pre (47.51 ± 4.12%) vs Stim on (48.52 ± 4.95%) $p > 0.05$
Figure 5 H	$n = 6$ for each group	passed	paired t test (two-tailed)	t (5) = 0.1788<br>t (5) = 0.8644<br>t (5) = 1.051<br>t (5) = 1.254<br>t (5) = 0.6108	Pre delta (59.83 ±4.76%) vs Post delta (60.24 ±4.52 %) $p > 0.05$<br>Pre theta (24.91 ± 3.37%) vs Post theta (21.94 ±1.82 %) $p > 0.05$<br>Pre alpha (9.40 ± 1.26%) vs Post alpha (11.58 ±2.63 %) $p > 0.05$<br>Pre beta (3.74 ± 0.42%) vs Post beta (4.30 ±0.65 %) $p > 0.05$<br>Pre gamma (2.12 ± 0.37%) vs Post gamma (1.94 ± 0.33 %) $p > 0.05$
Figure 5 I	$n = 7$ for each group	passed	paired t test (two-tailed)	t (6) = 1.12<br>t (6) = 2.13	EYFP : Light off (204.1 ± 12.22 s) vs Light on (213.9 ± 10.92 s) $p > 0.05$<br>ChR2 : Light off (202.1 ± 10.81 s) vs Light on (211.9 ± 10.36 s) $p > 0.05$
Figure 5 J	$n = 7$ for each group	passed	paired t test (two-tailed)	t (6) =1.465<br>t (6) =3.712	EYFP : Light off (155.4 ±9.94 s)

					vs Light on (150.7 ±9.07 s) $p > 0.05$ ChR2 : Light off (153.6 ± 11.9 s) vs Light on (106.9 ± 9.87 s) $p < 0.01$
Figure 6 E	*n* = 7 for each group	passed	paired t test (two-tailed)	t (6) = 1.274 t (6) = 0.4561	EYPF-saline (214.9 ± 11.7 s) vs EYPF-CNO (220.6 ± 13.52 s) $p > 0.05$ hM4Di-saline (205.4 ±12.91 s) vs hM4Di-CNO (211.1 ±13.68 s) $p > 0.05$
Figure 6 F	*n* = 7 for each group	passed	paired t test (two-tailed)	t (6) = 1.436 t (6) = 4.205	EYPF-saline (192.6 ±11.89 s) vs EYPF-CNO (208.7 ± 17.66 s) $p > 0.05$ hM4Di-saline (178.9 ± 13.33 s) vs hM4Di-CNO (216.4 ± 17.67 s) $p < 0.01$
